# Postoperative sinus node dysfunctions—maybe not always the surgeon should to be blamed…

**DOI:** 10.1093/icvts/ivad009

**Published:** 2023-01-18

**Authors:** Katarzyna Januszewska

**Affiliations:** Polish Mother’s Memorial Hospital - Research Institute, Rzgowska 281/289, 93-338 Lodz, Poland

**Keywords:** Congenital heart defects, Epicardial mapping, Sinus node dysfunction

Dr. Misier *et al.* [1] present 3 children with simple congenital heart defects and preoperative sinus rhythm who underwent intraoperative electrophysiological epicardial mapping which discovered alterations regarding the sinoatrial node (SAN) exit sites as well as intraartial conduction. All these 3 children developed postoperative sinus node dysfunction (like sinus bradycardia, ectopic atrial rhythm, etc.). The message of the study is that the pre-existing alterations in SAN exit sites and atrial conduction disorder predispose children with congenital heart defects for early postoperative sinus node disorders. This is a very interesting study, however, to drive such a conclusion a control group without preoperative alterations is needed. In the current clinical praxis, it seems to be obvious, that in a child with preoperative normal sinus rhythm, the postoperative rhythm disorders are most likely due to the surgical procedure, like injury of the sinus node or its arterial supply, damage of the conduction pathways or at least temporary disorders due to hypoxaemia, hypoperfusion, oedema, etc. It is very nice to read, that not always the surgeon should to be blamed for the postoperative arrhythmias, but the evidence provided by the study is in my opinion not convincing. First of all, to drive such a conclusion suggesting a causal relationship, the control group is needed. It would be interesting to know if the 20 remaining patients from the FANTASIA study (of which the current study was a part of) did demonstrate any postoperative sinus node dysfunction. This is a very interesting topic and because of this I would like to encourage the authors to conduct such a study (as I understood the data are already collected in the FANTASIA study). The results of the study are interesting from the perspective of the other studies as well. According to one of the investigations, the presence of 2 distinct functionally active sinoatrial nodal pacemakers in the human heart (as well as in other mammalians), which dominate at distinct ranges of heart rates and are located at the 2 orifices of the venae cavae, seems to be normal [2]. In the study of Dr. Misier *et al.*, inferior or multiple SAN exit sites were identified, which does not mean that superior SAN was not present or damaged. It can mean that, in this specific situation (e.g. under the anaesthesia), the inferior SAN was just active at that moment. Anyway, the study hypothesis is an inspiring idea and I would like to encourage the authors to develop this idea.
